# Network Physiology of Exercise: Vision and Perspectives

**DOI:** 10.3389/fphys.2020.611550

**Published:** 2020-12-11

**Authors:** Natàlia Balagué, Robert Hristovski, Maricarmen Almarcha, Sergi Garcia-Retortillo, Plamen Ch. Ivanov

**Affiliations:** ^1^Complex Systems in Sport, INEFC Universitat de Barcelona (UB), Barcelona, Spain; ^2^Faculty of Physical Education, Sport and Health, Ss. Cyril and Methodius University, Skopje, North Macedonia; ^3^University School of Health and Sport (EUSES), University of Girona, Girona, Spain; ^4^Keck Laboratory for Network Physiology, Department of Physics, Boston University, Boston, MA, United States; ^5^Harvard Medical School and Division of Sleep Medicine, Brigham and Women’s Hospital, Boston, MA, United States; ^6^Institute of Solid State Physics, Bulgarian Academy of Sciences, Sofia, Bulgaria

**Keywords:** network physiology, exercise physiology, sport sciences, fitness, sports performance, sports medicine, dynamic networks, complex systems

## Abstract

The basic theoretical assumptions of Exercise Physiology and its research directions, strongly influenced by reductionism, may hamper the full potential of basic science investigations, and various practical applications to sports performance and exercise as medicine. The aim of this perspective and programmatic article is to: (i) revise the current paradigm of Exercise Physiology and related research on the basis of principles and empirical findings in the new emerging field of Network Physiology and Complex Systems Science; (ii) initiate a new area in Exercise and Sport Science, Network Physiology of Exercise (NPE), with focus on basic laws of interactions and principles of coordination and integration among diverse physiological systems across spatio-temporal scales (from the sub-cellular level to the entire organism), to understand how physiological states and functions emerge, and to improve the efficacy of exercise in health and sport performance; and (iii) to create a forum for developing new research methodologies applicable to the new NPE field, to infer and quantify nonlinear dynamic forms of coupling among diverse systems and establish basic principles of coordination and network organization of physiological systems. Here, we present a programmatic approach for future research directions and potential practical applications. By focusing on research efforts to improve the knowledge about nested dynamics of vertical network interactions, and particularly, the horizontal integration of key organ systems during exercise, NPE may enrich Basic Physiology and diverse fields like Exercise and Sports Physiology, Sports Medicine, Sports Rehabilitation, Sport Science or Training Science and improve the understanding of diverse exercise-related phenomena such as sports performance, fatigue, overtraining, or sport injuries.

## Introduction

The human organism comprises various multicomponent physiological systems that interact through various feedback mechanisms across a range of nonlinear feedback mechanisms, operating across spatio-temporal scales to generate complex transient dynamics that continuously adapt to intrinsic and external perturbations. The traditional reductionist approach, employed to investigate physiological systems and their regulatory mechanisms based on classical cybernetics, is insufficient to provide a comprehensive understanding of the structure and dynamics of individual systems and how systems and subsystems coordinate their dynamics across various levels of interaction to generate integrated functions at the organism level. Synchronization and integration among physiological systems is essential to generate distinct physiological states (e.g., sleep and wake, rest and exercise, health, and disease) and, therefore, unraveling the underlying principles of physiological systems integration as a network is crucial to understand how various physiological functions emerge as a result of interaction among such systems. Recent research has shown that physiologic states emerge as a result of a very particular network organization, network topology, and network dynamics of interaction among systems and subsystems (Ivanov and Bartsch, 2014; Ivanov et al., 2017). This dynamic network-based approach to human physiology has the potential to broaden the scope and provide more comprehensive framework of investigations also in the field of Exercise and Sports Physiology and can help address fundamental questions: (i) How muscle fibers within muscle groups and different muscle groups in the human body coordinate their activation during exercise and how this coordination is affected by fatigue? (ii) How organ systems communicate and coordinate as a network to satisfy certain task demands? (iii) How training modifies physiological systems coordination at multiple spatio-temporal scales? (iv) Which are the coordination-related improvements produced by exercise and what are the associated risk factors, the effects on health, and prevention and treatment of chronic diseases? Addressing these questions would have important implications for both Basic Physiology and would open a new frontier of investigations in Exercise and Sports Physiology, Sports Medicine, Sports Rehabilitation, Sports Sciences, and all their different specialties and subfields.

There is an epistemological gap in the scientific research of physiological systems in the field of Sports and Exercise. The prevalent approach is the mechanistic one (Machamer et al., 2000; Bechtel and Richardson, 2010), which aims to uncover the physiological mechanisms responsible for the phenomena under consideration by reducing complex multicomponent system on their parts. The newer, and hence, less developed line of research in the field of Sports and Exercise Physiology is the complex dynamic systems approach, which focuses on the systems complex dynamics with the aim of discovering and formulating general principles on which biological system functionality is based. A crucial distinction between the reductionist and integrative approaches is how they treat the dynamics of biological systems.

Although integrative physiology recognizes the importance of interconnectivity across physiological systems (Sieck, 2017), its research methodologies have traditionally focused on statistical inference of static associations of vertical bottom-up mechanistic causation from the sub-cellular and cellular level to tissue, organ or organism level, and the regulatory functions that govern our physiological state and our health (Head, 2020). The natural evolution of Exercise Physiology toward Genetics and Molecular Biology, has emphasized the collection of integrated analytical approaches that composes the OMICS and contribute to the field of Molecular Exercise Physiology (Wackerhage, 2014; Gomes et al., 2019). As a consequence, there is a wide uncharted territory in research and absence of knowledge in the direction of dynamic characteristics of such vertical integration, as well as the horizontal integration of key organ systems network interactions.

A new field, Network Physiology, has recently emerged to fill in this gap (Bashan et al., 2012; Bartsch and Ivanov, 2014; Ivanov and Bartsch, 2014; Bartsch et al., 2015; Liu et al., 2015b; Ivanov et al., 2016, 2017) and to address the fundamental question of how physiological systems and subsystems coordinate, synchronize, and integrate their dynamics to optimize functions at the organism level and to maintain health. It aims at uncovering the biological dynamic mechanisms (Chen et al., 2006; Ivanov et al., 2009; Bechtel and Abrahamsen, 2010; Bartsch et al., 2012) since it satisfies both the mechanistic requirement of structure and localization (e.g., nodes and edges/links in dynamic networks may represent localized integrated organ systems, subsystems, localized components or processes, and interactions among them across various levels in the human organism) and the requirement of dynamical invariance and generality that is enabled by dynamical systems approach (Meyer, 2020). Organ interactions are essential to produce health, and uncovering the underlying mechanisms of physiologic network dynamics and control is crucial to fully understand the effects of exercise on health, treatment of disease and sports performance.

Disrupting organ communications and their dynamic coordination as a network can lead to dysfunction in individual systems or the collapse of the entire organism during exercise (e.g., fatigue, task failure, and injuries; Hristovski and Balagué, 2010; Balagué et al., 2014b; Vázquez et al., 2016; Pol et al., 2018). Thus, in addition to the traditional approach in biology and physiology that defines health and disease states through structural, dynamic, and regulatory changes in individual systems, the new conceptual framework of Network Physiology focuses on the coordination and network interactions among systems as a hallmark of physiological state and function.

In dynamic networks of physiological interactions, the networks links represent interactions and synchronization between systems and subsystems, and exhibit transient time-varying characteristics (Bartsch et al., 2014; Lin et al., 2016, 2020). A key question is how physiological states and functions emerge out of the collective network dynamics of integrated systems (Bartsch et al., 2015). While network structure may play an important role in generating various states and functions, different global behaviors could emerge due to temporal changes in the functional form of physiologic interactions without reorganization in network topology. This poses new challenges to develop generalized methodology adequate to quantify complex dynamics of networks, where network nodes represent dynamic components of the system and network links reflect different forms of coupling that may change over a range of timescales.

Each physiological system exhibits complex dynamics with a remarkable amount of distinct rhythms that are coupled and coordinated over several magnitudes of timescales. Specifically, previous research has identified the presence of complex temporal organization and long-range power law correlations in the signal output of physiological systems and how temporal characteristics change with transition across physiological states (rest and exercise, sleep and wake, sleep stages, and circadian phases) in the cardiovascular system (Ivanov et al., 1996, 1999a,b, 2001, 2004; Amaral et al., 1998; Ashkenazy et al., 2001; Bernaola-Galván et al., 2001; Goldberger et al., 2002; Kantelhardt et al., 2002; Karasik et al., 2002; Ivanov et al., 2007), the respiratory system (Cernelc et al., 2002; Peng et al., 2002; Suki et al., 2003, 2005), the brain (Linkenkaer-Hansen et al., 2001; Lo et al., 2002, 2004; Beggs and Plenz, 2003; Angelini et al., 2004; Poil et al., 2008; Chialvo, 2010; Schumann et al., 2010; Lombardi, et al., 2012, 2020a,b; Palva et al., 2013; Arcangelis et al., 2014; Liu et al., 2015a), in gait dynamics (Hausdorff et al., 1995, 1997, 2001; Ashkenazi et al., 2002), in wrist motion (Hu et al., 2004; Ivanov et al., 2007), and in the musculo-skeletal system (Kerkman et al., 2018, 2020; Garcia-Retortillo et al., 2020). Investigations in Basic Physiology through the prism of system dynamics have revealed fundamental scale-invariant characteristics that are universal across subjects that encompass a broad range of timescales, indicating the presence of multi-scale mechanisms of physiologic regulation (Ivanov et al., 1998, 2004; Hausdorff et al., 2001; Lo et al., 2002; Kantelhardt et al., 2003; Schumann et al., 2010). Furthermore, every physiological system functions as a dynamic node interacting with other systems through multiple parallel links on a wide range of frequency domains (Bartsch et al., 2012, 2014; Liu et al., 2015b; Lin et al., 2016). The links within a given network adjust the intensity of information transfer (i.e., link strength), so that certain links play the role of major mediators of the interaction between two systems, while other links may present an auxiliary supporting function, thus leading to hierarchically structured organization and profiles of network links strength that are specific for each physiological state. Every physiological state under health or disease (e.g., wake and sleep, sleep stages, rest and exercise) is achieved by means of highly detailed adjustments in the multiple link interactions between dynamical systems – for instance, while during deep sleep brain-heart interactions are characterized by links with identical strength across frequency domains, high-frequency links are the main mediators of brain-heart interactions during wake (Bartsch et al., 2015); furthermore, during the same physiological state, the interaction between different pairs of organ systems can be mediated by dominant links in different frequency domains (Bartsch et al., 2015; Liu et al., 2015b; Ivanov et al., 2017).

In recent years, the Network Physiology framework has been utilized in various fields of basic Physiology and Clinical Medicine, including multiple organ failure and sepsis in critically ill patients (Asada et al., 2016; Moorman et al., 2016), neonatal intensive care (Lavanga et al., 2020; Lucchini et al., 2020), liver disease (Tan et al., 2020), epilepsy and neurological disorders (Lin et al., 2020), diabetes and obesity (Podobnik et al., 2020; Prats-Puig et al., 2020), cancer (Liu et al., 2020), or psychiatry (Bolton et al., 2020), and has the potential for broad applications in the field of Exercise Physiology and Sports Medicine to uncover how the key physiological systems interact pairwise, that is, which links are the major mediators in a given network and how these links adjust their strength with accumulation of fatigue, after a training intervention, or in response to a certain pathological condition (e.g., musculo-skeletal injury and neurodegenerative disease).

The aim of this article is to provide a vision and a new programmatic framework for basic research and practical applications of Network Physiology to Exercise and Sports Science. We propose a new theoretical framework for investigations in Exercise Physiology based on principles and approaches based on Network Physiology and Complex Systems Science. We discuss early works and provide a vision for future research directions in a new emerging field, Network Physiology of Exercise (NPE), utilizing examples of exercise prescription for health and disease, where we focus on exercise recommendations for healthy population and clinical patients (also relevant for sports performance), and we point toward the practical perspectives and future developments in NPE.

## Exercise Physiology and Network Physiology of Exercise: Contrasting Approaches

**Figure 1:**
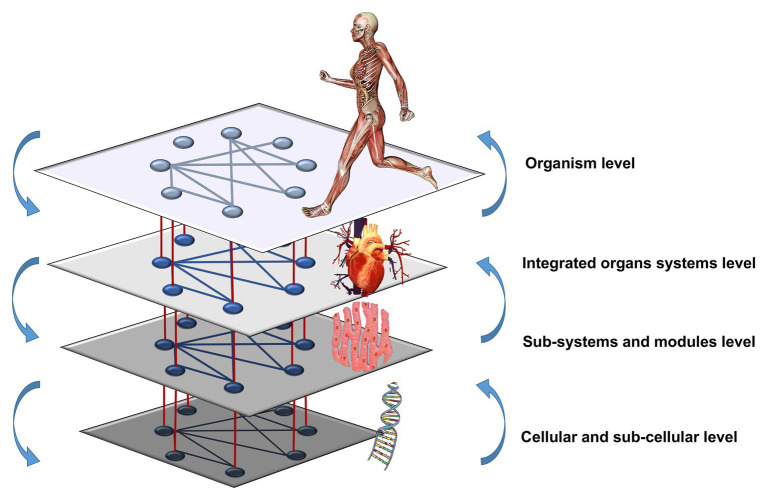
Schematic diagram for the vision on Network Physiology of Exercise. Hierarchically organized physiological network levels interact both horizontally and vertically through circular causality.

Figure 1 shows a schematic diagram for the vision of NPE. A hierarchical organization of embedded networks into networks (genetic, tissue, organ, systemic, etc. networks) interact dynamically (horizontally and vertically). Each of them has its own regulatory mechanisms but mutually interact and operate at different levels and timescales (Thompson and Varela, 2001; Tarasov, 2019). Upper and lower network levels are related through circular causality: bottom-up, new components (cells, tissues, organs, etc.), and their properties emerge through a self-organizing process. Top-down, the higher levels constrain the lower ones (Noble et al., 2019; Tarasov, 2019).

The network science-based vision of NPE is in contrast with that of current Exercise Physiology, strongly influenced by reductionism. In this section, we contrast the theoretical assumptions of both approaches focusing on some main misconceptions that affect the understanding of diverse exercise-related phenomena: the adaptive properties of the human organism, the understanding of the physiological states of health and fitness, the objective and principles of exercise training, the assessment of the physiological status, and the role of exercise professionals and users/patients. Table 1 summarizes the main traits.

**Table 1:** Contrast between theoretical and practical assumptions of training under Exercise Physiology and Network Physiology of Exercise (NPE) perspectives.

Assumptions	Exercise Physiology	Network Physiology of Exercise
Scientific approach	Reductionism Cybernetic control theory	Network theory Dynamic systems theory
Conception of organism	Machine	Complex adaptive system
Relations	Linear and dose-response	Nonlinear dynamic interactions
Dynamics	Component-dominant	Interaction-dominant
Adaptive properties	Homeostasis	Homeodynamics, spontaneous synergies, degeneracy, and pleiotropy
Training goal (development of)	performance attributes (aerobic and strength)	Functional diversity potential and somatic awareness
Training programs	Pre-defined (universal recipes)	Contextually sensitive (personalized in space and time)
Training and rest dosification	Progressive volume and intensity	Balancing requirements and immediate inner capabilities
Training methodology	Prescription-based	Self-regulation oriented
Training tasks	Imposed aerobic and strength exercises	Contextually meaningful (motivating and co-designed)
Type of exercises	Repetitions	Variations
Evaluation	Cardiopulmonary and strength tests	Connectivity tests
Monitoring	Dominantly objective and external devices	Dominantly subjective and somatic awareness
Professionals/patients and athletes	Prescribers/executers	Co-designers of the process

### From Reductionism to Dynamic Networks Integrative Approach

Exercise Physiology, the most influential discipline in exercise and sports training, has remained resistant to the introduction of the science of complex systems in biology (Hristovski et al., 2014; Balagué et al., 2016; Pol et al., 2020). Reductionism has dominated the research and has shaped the way of thinking of exercise professionals. To understand any physiological phenomenon, reductionism breaks it down into increasingly smaller parts with the help of technological advances: organisms are dissected, cells isolated, etc. Its influence explains the persistent search for cellular, biochemical, and genetic mechanisms, and the causes of macroscopic phenomena like exercise-induced fatigue, strength or aerobic capacity, etc. For instance, even if there is strong scientific evidence that lactate and other exercise metabolites do not limit exercise performance, a good amount of current research continues to investigate this topic (Hristovski et al., 2014). The fragmentation of fitness in dimensions (endurance, strength, velocity, etc.) and sub-dimensions (maximal strength, explosive strength, etc.), and the isolation of muscle groups with training purposes are also common practices derived from reductionism.

Instead of the usually assumed causal bottom-up effects from micro‐ to macro-structures and processes, the NPE approach, applying the principle of circular causality, assumes a bottom-up/top-down relationship between micro and macro-components (Noble et al., 2019; Tarasov, 2019). For instance, genes affect organ functions and organ functions constrain gene expression.

### Complex Adaptive Systems: Component vs. Interaction-Dominant Dynamics

The understanding of human organisms as complex adaptive systems (CAS), instead of complicated systems (e.g., machines or technical devices), has several theoretical and practical implications on exercise prescription. Contrary to what is usually assumed in Exercise Physiology, in CAS, the behavior emerges from the interaction among components and cannot be explained (or reduced) to any single component. Studying elements of complex systems in isolation is by definition incomplete, as interactions generate novel information that determines the future of elements and thus of the system itself (Gershenson and Fernández, 2012). This dominant interaction dynamics, in contrast with component-dominant dynamics (Van Orden et al., 2003), has been emphasized by several authors (Delignieres and Marmelat, 2012; Vázquez et al., 2016; Almurad et al., 2018). It means that the behavior of CAS cannot be simply explained through the variability of any single component, process, or local mechanism. For instance, exercise physiologists cannot rely on critical quantitative endpoints in cardiovascular, respiratory, metabolic, or neuromuscular systems to explain the limits of performance (Noakes, 2000; Venhorst et al., 2018; Pol et al., 2020) and should reformulate their research hypothesis accordingly.

### Complex Adaptive Systems Interact Dynamically, Nonlinearly, and Co-adaptively With the Environment

This means that their interactions change in time, and not only quantitatively but also qualitatively. For this reason, there are neither clearly separable cause-effect or dose-response relations among components nor time-invariant mechanisms and regulation profiles. In Exercise Physiology, the integrative functions are studied within the framework of the traditional control theory, and concepts such as homeostasis, feedback loops and central programmers are usually evoked to describe system regulation during exercise (Lambert, 2005). The behavior predictions of this “engineering” approach are linear, i.e., proportional between inputs and outputs. The basic assumption is that of time-invariant encapsulated processes and regulation profiles. As long as one deals with conceptual, i.e., verbal, descriptive modeling, this approach based on explicit feedback loops seems fine, but when trying to model mathematically more than a couple of interlinked components together, then the system rapidly becomes impossible to treat in terms of explicit feedback circuits and presents serious prediction problems.

In NPE, as exercising individuals interact nonlinearly with their environment, the exercising unit is the performer-environment system (Araújo and Davids, 2016). This means that the individual adaptive responses to exercise are unique and contextually dependent. Feedback homeostatic mechanisms are replaced by the concept of homeodynamics or dynamic stability, i.e., a constantly changing interrelatedness of body components and processes while an overall equilibrium is maintained (Bassingthwaighte et al., 1995).

As nonlinear and history-dependent systems, physiological networks present hysteresis, a phenomenon that explains the delay in a system’s recovery of its initial state after a perturbation (Hristovski et al., 2010, 2014; Montull et al., 2020). The study of this phenomenon, a hallmark of complex systems, has revealed the limitations of the widely used linear and proportional regulation models in Exercise Physiology (Abrantes et al., 2012), and has shown the excessively simplified assumptions and artificially created contexts (e.g., in fitness testing). The same exercise perturbation (external load) does not have the same impact (internal load; Fullagar et al., 2019), depending on the previous exercise, the state of the network, and other contextual differences. The area of hysteresis has recently been pinpointed as a new non-invasive marker of exercise stress and tolerance to test the state of the network (Montull et al., 2020).

### From Homeostasis of Individual Systems to Emergence and Self-Organization at the Organism Level

Qualitative changes occurring in CAS are the product of a widely ignored property in Exercise Physiology: self-organization. Physiological components and processes acting at multiple levels (from molecules to systems) are spontaneously coupled and there is no need for a template or internal (nor external) programmer to rule the relations. That is, our physiological systems, organs, tissues, and cells change spontaneously through their morphology and function, constrained by evolved genes and their expression, chemical species, natural and social environment, etc. (Sturmberg, 2019). Then, exercise regulation is better understood as a complex, goal-directed, and context-dependent dynamic mechanism adapting to continuous emerging organismic and environmental constraints. In such a framework, nonlinear, i.e., non-proportional, individual physiological changes, and training effects are produced when exposing the organism to exercise and training loads (Hristovski et al., 2010). The authors explain how the same workload, which may promote positive adaptations in a specific context of a given system, may produce overtraining effects and emergent behaviors in another context through coupling, feedbacks, and network interactions.

### From Microscopic Functions to Macroscopic Behaviors

**Figure 2:**
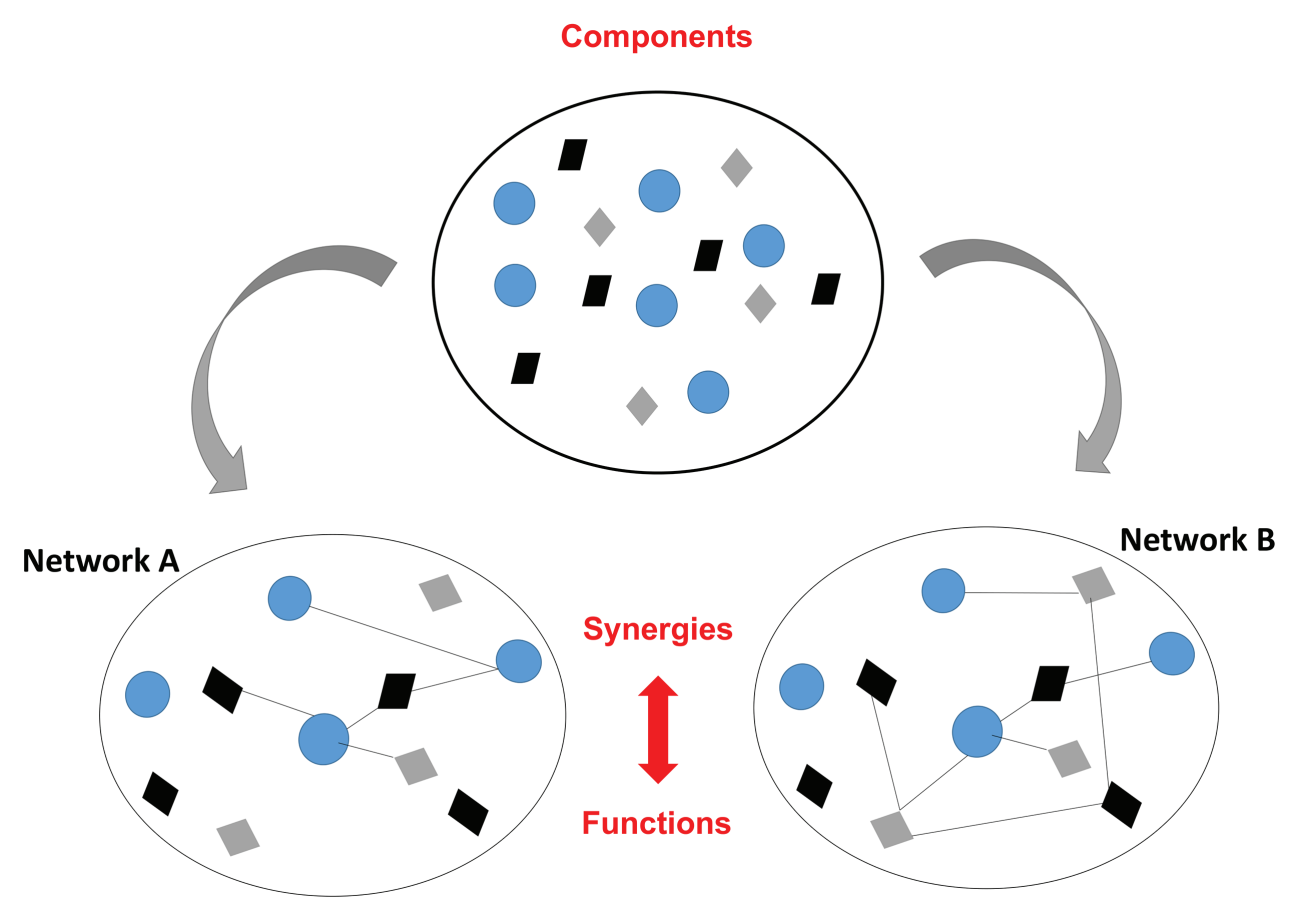
Formation of synergies across levels to satisfy task goals during exercise. Up: representation of different types of components of a certain physiological network. Down: system components, coupled by links, reorganize to form different structural and functional synergies **(A,B)** to achieve the same task goal under changing constraints. Network components are represented by symbols and their links through black lines.

The term “training”, understood as the process of learning/acquiring specific skills has been recently proposed to be replaced by the term of “synergizing”: combining or working together to be more effective (Pol et al., 2020). Synergies are the spontaneously formed structural and functional couplings among components and processes to achieve the main goal in health: keeping the homeodynamics or dynamic stability (Riley et al., 2011; Kelso, 2017; Latash, 2019; Liu et al., 2019). During exercise, synergies operating at diverse scales are continuously re-organized, allowing the reciprocal compensation of components and processes to satisfy task goals. They have circular causal relations with components; that is, components form synergies and those synergies, in turn, govern the components’ behavior (Noble et al., 2019; Tarasov, 2019; see Figure 2). As shown in the figure, synergies manifest the property of degeneracy; different components can produce the same function and different synergies may be activated to achieve the same task goal (Edelman and Gally, 2001; Latash, 2019). For instance, different motor units cooperate and adjust their activation over several timescales to perform an effective or functional motor action over time. The self-assembled, adaptive interactions of CAS underpin also another robustness-enabling property: pleiotropy or multifunctionality, that is, the same components may be assembled to produce multiple functions; for instance, the skeletal muscle has contractile, immunological, and endocrine functions (Pedersen and Febbraio, 2012; Sallam and Laher, 2016). Such properties enable CAS to switch between diverse coordinative states and maintain a metastable dynamic (Bovier and Den Hollander, 2016).

### From Developing Isolated Performance Markers/Dimensions to Increased Diversity Potential of Network-Based Measures

Physical fitness is defined as the ability to carry out daily tasks with vigor and alertness, which is better achieved by developing fitness attributes and producing a substantial increase in caloric requirements over resting energy expenditure (American College of Sports Medicine, 2009). In Exercise Physiology, fitness attributes are mostly associated with strength and conditioning. In contrast to this assumption, a new definition of fitness, inspired on theories of biological evolution, has been recently introduced (Pol et al., 2020). The authors sustain that the fittest is not necessarily the fastest or strongest but the most diverse. Accordingly, from a NPE perspective, fitness is defined as the ability to survive in a broad range of contexts, that is, to adapt to socio-psycho-biological challenges (Sturmberg, 2019). In neurobiological systems, dynamic stability, which means survival over long timescales, can only be achieved through a continuous process of complexification, i.e., diversification and specialization (Pross, 2016). Although higher strength or endurance levels imply higher functional (i.e., good variance) diversity, this property cannot be just reduced to these attributes because it embraces multiple dimensions. Furthermore, it may be attained through different processes and in different ways according to the degeneracy property (Edelman and Gally, 2001; Pol et al., 2020).

For instance, a gymnast has more chances to become dynamically stable (i.e., surviving in the competition) by specializing and diversifying the elements of their floor routine. This subsumes diverse functional synergies (reciprocal compensations) coping with diverse and challenging environments (mainly represented by the opponents). In sports, this process of complexification is defined by the athlete’s functional diversity/unpredictability potential (Hristovski, 2017; Hristovski and Balagué, 2020), being unpredictability, a relational variable that arises within the performer-environment system and cannot be reduced to the development of strength and conditioning. Accordingly, a change of focus is proposed in fitness programs. Gaining functional diversity, instead of developing aerobic capacity and muscle strength, is the main aim. Diversity can be developed in many ways, not simply through aerobic and strength training, and it is better achieved through varied, non-repetitive training stimulus (Pol et al., 2020).

**Figure 3:**
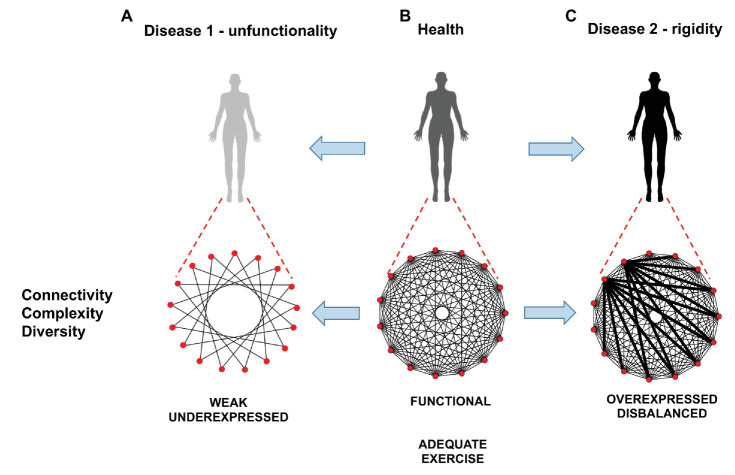
Effects of exercise on functional physiological network connectivity, complexity, diversity. From left to right: **(A)** weak or underexpressed connectivity, corresponding to unfunctional state, **(B):** functional connectivity, corresponding to healthy, fit state, and **(C)** underexpressed connectivity, corresponding to unfunctional state. Red nodes represent the different physiological network components and the links among them the couplings. The strength of the couplings is illustrated by the thickness of the links.

Figure 3 represents how exercise may modulate communications among physiological systems across levels and timescales leading to changes in functional network connectivity, complexity, and diversity potential of the physiological systems and subsystems promoting health and performance. Physiological organ systems and their components operate at diverse scales (Bashan et al., 2012; Ivanov and Bartsch, 2014; Gosak et al., 2018), modify the number and strength of time-varying (Bartsch et al., 2012, 2014; Bartsch and Ivanov, 2014) couplings, reorganize and reconnect creating new synergies essential to generate distinct physiological states and functions at the organism level (Bartsch et al., 2015; Liu et al., 2015a) and to respond to various task demands or training workloads, thus contributing to the homeodynamics (Balagué et al., 2016; Garcia-Retortillo et al., 2019a). Recent studies have demonstrated that basic physiologic states (wake/sleep and sleep stages) are associated with specific physiological network topology and hierarchical structure of interactions among key organ systems and that physiological networks reorganize with transitions across states to facilitate change in physiological function (Liu et al., 2015b; Ivanov et al., 2017; Rizzo et al., 2020). In the context of exercise, overcompensation response to training (Verkhoshansky and Siff, 2009) may be reflected by increase in coupling intensity and overexpressed connectivity among physiological systems and subsystems leading to reorganization in physiological network structure and dynamics (Figure 3B). In contrast, weak or underexpressed connectivity (Figure 3A) could be hypothetically associated to sedentarism and injuries (Pol et al., 2018), while excessive exercise could be associated with a transitory underexpression of coupling network connectivity (i.e., imbalance: some processes are overexpressed and others underexpressed; Figure 3C). An example of such imbalance is the rigidity and reduction of diversity potential that accompanies exercise-induced fatigue (Vázquez et al., 2016). In a similar way, some pathological conditions (e.g., neuro-muscular disorders) could increase the density and/or strength of interactions among certain physiological rhythms, pushing the system toward a rigid order and reducing the robustness and adaptability to environmental changes (Ivanov et al., 1998, 2001; Stergiou et al., 2006; Stergiou and Decker, 2011).

### Health: From Additive Static Systems to Emergent States and Functions From Dynamic Network Interactions

The WHO defines health as a state of complete physical, mental and social well-being and not merely the absence of disease or infirmity (World Health Organization, 2019). This additive definition, including the physical, mental and social dimensions, contrasts with that of Sturmberg (2019) that defines health as an adaptive, subjective, emergent state of the whole person that arises from hierarchical network interactions between different levels: ecological, social, physiological, genetic, etc. (see Figure 1). From this perspective, health is the result of dynamic interdependencies between the external environment and the internal physiology. According to the authors, it refers to adapting to socio-psycho-biological challenges and can occur in both absence or presence of objective disease. Due to its experiential and dynamic nature it may change in response to somatic conditions, social connectedness, emotional feelings and semiotic (or sense-making; Sturmberg, 2019). This means that a healthy state experience can be achieved in different ways and that there is no unique health state prototype. Thus, effective care must combine strategies centered on the person with those from the NPE perspective.

### From Universal Training Program Recipes to Contextually Sensitive Training Criteria

On the basis of current research evidence and simplified assumptions of Exercise Physiology, universal training programs are prescribed for healthy and clinical populations (American College of Sports Medicine, 2009; World Health Organization, 2019). These one-size-fits-all recommendations assume the existence of decontextualized realities (Jones et al., 2017) and ideal or prototypic fitness and health states. However, maximization or minimization of CAS is very hard to define, and consequently to measure and prove due to their context-dependency. Thus, it is recommendable to focus on individual optimality, defined in space and time, and as such, evolving dynamically. Hence, one can speak about larger or smaller adequacy of interventions (see e.g., Chandler, 2018).

The application of the training principles of individualization, specificity, adaptivity, and periodization are a good example of excessively simplified assumptions of Exercise Physiology: (a) The exercise personalization is based on the objective evaluation of a patient’s baseline physiological status (American Thoracic Society and American College of Chest Physicians, 2003; Myers et al., 2015); (b) there are specific physiological adaptations to different types of exercise; (c) the adaptation to different intensities, durations, and training frequencies are based on a dose-response relationship, and (d) periodization subsumes the progressive overloading, adequate rest and recovery to maximize the adaptive response (Lorenz et al., 2010).

The principle of training periodization is particularly controversial in pre-established training programs (Kiely, 2018). The assumption that exercise sequencing and scheduling should be based on mechanical training stress ignores, for instance, the neuro-endocrine and bio-chemical consequences of the psycho-emotional stress that overlay training stimuli. This explains why there is no one-to-one mapping between training dose and training effects. The same load that promotes adaptivity may produce overtraining when the context changes (Hristovski et al., 2010). As many personal and environmental constraints change unexpectedly, a long-term training periodization cannot be sufficiently responsive to flexibly adjust to continuous and unpredictable co-adaptive performer-environment processes. In such variable contexts, it seems more adequate that the training process itself, and not the training program, leads and shapes the personalized workload adjustments (Orth et al., 2019). Although several nonlinear periodized prescriptions have proved their higher efficacy, compared to traditional ones, in improving cardiorespiratory fitness (and other important clinical outcomes) for different clinical populations (Jones et al., 2008; Klijn et al., 2013), a further progress in the direction of personalized adjustments is warranted.

Methodological criteria derived from NPE principles (e.g., stability, instability, constraints, change of state, etc.) and defined at multiple levels (Hristovski et al., 2014; Pol et al., 2020) can be used to personalize and coadapt fitness programs avoiding long-term periodization. They compress without fragmenting the huge complexity of dimensions (physiological, psychological, social, etc.), levels, and timescales involved in exercise training (Garcia-Retortillo et al., 2020; Pol et al., 2020).

### From Prescribing Exercise Programs to Co-adapting Processes

As long as the training objective is focused on the diversification of complex physiological networks, and not on attaining pre-established fitness outcomes, the training process requires a redefinition of the role of the agents involved in it. Under the NPE perspective, exercise professionals and users/patients/athletes constitute a learning system, in which the exercise professional is not only the manager of the training environment but also a learning component (Orth et al., 2019). Since actions emerge from the performer-environment interaction, the continuous adjustments of workloads and constraints needs the active participation of the users/patients/athletes, which are expected to be not mere executers of the program but their codesigners (Pol et al., 2020). This active involvement of users/patients/athletes in the process supposes a collateral process of developing their somatic awareness (Pol et al., 2018). This skill is essential for capturing personal and environmental constraints changing at fast timescales (e.g., fatigue state, psycho-emotional state; climate, etc.; Balagué et al., 2019) and adapting workloads accordingly. The implementation of adequate subjective assessment tools with pedagogical and exploratory purposes can assist the exercise professional and contribute substantially to healthcare (Pol et al., 2018; Sturmberg, 2019). The implication of the user/patient/athlete as co-designer of the intervention is also crucial to increase their adherence, a key factor for the success of any training program. The exercise professional, in turn, should be mostly focused on selecting and providing adapted, varied, and sufficiently challenging proposals to develop the diversification potential of users/patients/athletes.

### From Standardized Tests to Testing Methodologies Based on Functional Diversity

Cardiopulmonary exercise testing is the common assessment of choice for the accurate quantification not only of cardiorespiratory fitness but also for an integrative evaluation of the physiological response to exercise. Additional functional insights are also recommended through assessments of muscular strength, muscular endurance, and balance (American College of Sports Medicine, 2009).

The extracted quantitative variables from cardiopulmonary exercise testing (e.g., VO_2max_, ventilatory thresholds, etc.) are unable to capture the changes in the network dynamics produced by exercise and training. New methodologies based on continuous and synchronous recordings of multiple physiological parameters are needed to assess the qualitative network reorganization and compensatory synergies accompanying the exercise perturbations.

The assessment of correlation properties in the series produced by physiological parameters allows us to determine the possible alterations of complexity, either toward disorder (in which case correlations tend to extinguish in the series) or toward rigid order (in which case correlations tend to increase). From this point of view, complexity is conceived as an optimal compromise between order and disorder. This dynamic is characterized by long-range correlated series (1/f fluctuations; Delignieres and Marmelat, 2012), which can be detected using fractal analysis methods (Peng et al., 1995). The loss of complexity can be produced either by a decrease of the density of interactions between components or by the emergence of salient components that tend to dominate the overall dynamics (Figure 3). In the first case, the system derives toward randomness and disorder, and in the second toward rigidity. Complexity defines a fit state, characterized by robustness (or stability despite environmental perturbations) and adaptation to environmental changes. These relationships between complexity, robustness, adaptability, and health have been well illustrated by Goldberger et al. (2002) in the domain of heart diseases.

## Early Works and Future Research Directions in Network Physiology of Exercise

The new NPE assumptions contrasted in “Exercise Physiology and Network Physiology of Exercise. Contrasting Approaches” section change not only the understanding of exercise-related phenomena but also the research questions, the research methodologies and data analysis, the research interpretations, and their practical consequences in diverse fields of knowledge related to exercise and sport.

Considering the interaction of dominant dynamics of CAS, NPE research is focused on the vertical as well as the horizontal dynamic integration of networks (see Figure 1). The vertical integration assumes the study of couplings between lower and upper level networks (e.g., genomics and metabolomics networks with tissue networks, organic networks, etc., and vice versa), and the horizontal integration of the study of interactions among network components belonging to the same level (e.g., between organs: muscles, liver, lungs, and brain). To identify and quantify adequately vertical and horizontal dynamic interactions during exercise, new data analysis methodologies should be developed.

Methods applied to study physiological states non-related to exercise (e.g., wake, sleep, and disease) include: (i) cross-correlations of instantaneous phase increments – cerebral autoregulation and stroke (Chen et al., 2006), and migraine (Angelini et al., 2004); (ii) cross-correlations based on local and global detrending (Podobnik et al., 2009); (iii) automated phase synchronization technique – patterns of synchronous behavior between respiratory and cardiovascular systems (Bartsch et al., 2012); (iv) major component analysis of dynamic networks of physiologic organ interactions (Liu et al., 2015b), or (v) the time-delay stability technique ‐ a novel approach to infer and quantify interactions among diverse dynamical systems that studies the time delay with which bursts of activation in the output dynamics of a given physiological system are consistently followed, with constant time delay, by corresponding bursts in the signal output of other systems (Bashan et al., 2012; Ivanov and Bartsch, 2014; Bartsch et al., 2015).

Together with the aforementioned data science methodologies, other methods such as bivariate methods may be useful for analysis of stochastic processes with two macroscopically defined variables. More methods such as multivariate transfer entropy may be applied to interacting components at microscopic level and used for inferring more complex directed network structures (e.g., Novelli et al., 2019). For systems whose important dynamics can be determined by a few dominant oscillatory modes, the method of coupling functions (Stankovski et al., 2017) may provide a relevant determination of causal mechanisms of interaction among components. The Karhunen-Loeve decomposition (i.e., PCA; Carver et al., 2002; Hacken, 2006) may further play a methodological significant role not only in data dimension reduction but also in the explanation of the system’s functioning by determining the collective variables that enslave lower placed component processes. Recent theoretical work in this direction (Tarasov, 2019) opens the possibility to methodologically tackle temporally nested and long memory processes with power law behavior. Network measures such as clustering coefficients may provide important information about the structure of the networks since it is simultaneously a significant constraint on the dynamics within the network (Fagiolo, 2007). Future advances in treating problems discussed in this paper may require approaches based on mutually related multilayer and nested networks (Kivela et al., 2014). These approaches could provide rich information about the existence of coherently behaving communities within the network across its layers. This aspect is crucial for indept formal analysis and modeling of systems with nested interacting constraints that dwell on many spatial and timescales (Balagué et al., 2019; Tarasov, 2019).

Further research is required to develop new tests based on interorganic (horizontal) and multilevel (vertical) interactions, to complement the current assessment protocols used to evaluate fitness and the effectiveness of different exercise interventions. A better understanding of the physiological responses to exercise may assist exercise professionals with the selection of the most appropriate and safe exercise interventions. With the aim of uncovering the effects of exercise on the interactions among different physiological systems, future research programs within the framework of NPE should collect data simultaneously recorded from key organs including the brain, heart, or muscle during exercise. High spatio-temporal resolution instruments such as electroencephalography, electrocardiography, electromyography, accelerometry, or 3D MRI are promising tools to reveal important insights and successfully apply the aforementioned data analysis methods.

Early works on NPE have focused on improving the sensitivity to training and detraining of current fitness markers extracted from cardiopulmonary exercise tests. Cardiorespiratory coordination, a novel concept based on the co-variation among cardio-respiratory variables, has been introduced to assess changes produced by different training programs (Balagué et al., 2016; Garcia-Retortillo et al., 2019a), testing manipulations (Garcia-Retortillo et al., 2017, 2019b; Zebrowska et al., 2020), and nutritional interventions (Esquius et al., 2019). Cardiorespiratory coordination has been determined through a principal component analysis performed on time series of cardiovascular and respiratory variables registered during cardiorespiratory exercise testing (expired fraction of O_2_, expired fraction of CO_2_, ventilation, systolic blood pressure, diastolic blood pressure, and heart rate) and through the information entropy measures (information compression; Haken, 2006). During exercise, the interacting systems tend to attune their complexities in order to enhance their coordination. However, when the exercise demands increase, coordination among cardiorespiratory variables decrease. The main findings of this set of studies point toward a higher sensitivity and responsiveness of cardiorespiratory coordination to exercise effects compared to isolated cardiorespiratory outcomes, such as VO_2max_ and other gold standard markers of aerobic fitness. More recent research has investigated changes on cardiorespiratory coherence in response to hypoxic exposure and verified its dependence upon fitness status (Uryumtsev et al., 2020). These results indicate that strengthening connectivity among physiological systems provides optimal responses to hypoxic exposure and reflects the adaptive adjustment of the cardiorespiratory system in trained individuals. Furthermore, it has been recently demonstrated that the Network Physiology approach applied to exercise exhibits high sensitivity to quantify the performance of elite athletes participating at Olympic Games and to differentiate between fitness levels of those who win medals and those who do not (Pereira-Ferrero et al., 2019).

Perturbing the dynamic stability of the physiological network through exercise is crucial to test its health and fitness state because it provides a direct information about its adaptivity to changes. NPE methodologies may expand the knowledge on conditions of maladapted physiology provoked by excessive training load without adequate rest, such as overreaching or overtraining syndrome. Since these states result from a non-functional coupling between physiological subsystems (Kreher, 2016) and are not easily recognized through common physiological tests analyzing isolated outcomes (Meeusen et al., 2013), the tracking of changes on inter-organic interactions in response to training may contribute to develop new tools for early detection and prevention of such fatigue-related states. In fact, adaptivity is not necessarily linked to high maximal quantitative values achieved during cardiorespiratory exercise testing. For instance, an athlete affected by an overtraining syndrome will probably reach a high VO_2max_ but their adaptation to training workloads will be impaired. In contrast, the exploration of different features of the network dynamics like stability, instability, critical phenomena (enhancement of fluctuations and critical slowing down), or hysteresis behavior (Hristovski et al., 2014) may provide a rich information about the personal (in space and time) adaptive and qualitative fitness state. These features have been already tested during exercise using kinematic and psychophysiological variables (see Hristovski and Balagué, 2010; Balagué et al., 2014a, 2015; Garcia-Retortillo et al., 2015; Slapsinskaite et al., 2016; Vázquez et al., 2016; Montull et al., 2020) and the approach should be enlarged to physiological data.

Another maladaptive effect linked to a relevant research area that might benefit from NPE methodologies and principles is muscle injuries prevention. Previous research has related the susceptibility to suffer overuse musculo-skeletal injuries with abrupt changes on the connectivity of microinjuries and the concomitant motor coordination reconfigurations within the musculo-skeletal system (Pol et al., 2018). Therefore, given that interorganic reconfigurations might precede changes on a macroscopic level (Balagué et al., 2016; Garcia-Retortillo et al., 2017; e.g., macroinjury), new research programs are needed to develop novel tools capable of identifying and quantifying the interactions between structures and processes in the musculo-skeletal system and other relevant physiological systems. This would be of key importance to detect critical regions of constraints that increase the musculo-skeletal system susceptibility to suffer an injury (Pol et al., 2018).

In relation with the vertical dynamic interaction, future research should focus not only on bottom-up relations (e.g., from genes or exercise metabolites to organism performance) but also on top-down influences (e.g., from organs or motor actions to genes), including the effects of environmental constraints on physiological states, physiological systems, organs, or genes (Alabdulgader et al., 2018; Noble et al., 2019; Sturmberg, 2019). There may be specific social interaction of physiological effects that affects the vertical as well as horizontal integration. Another fruitful direction of research may be the phenomenon of strong anticipation (Stephen and Dixon, 2011) within the vertical or horizontal integrative realm.

Concretely, current main research directions of NPE aim to: (1) investigate how each organ system coordinate and couple its own distinct physiological rhythms, at a range of different frequency domains and over several magnitudes of timescales in response to exercise-induced fatigue and training load (intra-organ interactions), for instance, how different muscle fibers in a given muscle interact with each other and adjust their activation to create an optimal contraction; (2) explore how different muscles synchronize their activation to optimally perform a certain task (inter-muscular interactions); and (3) uncover the mechanisms underlying the synchronized activation among different brain areas and cortical rhythms and distinct key organ systems (e.g., cardiovascular, respiratory, and musculo-skeletal) during exercise (inter-organ interactions).

## Practical Perspectives

### Assessing Patients and Athletes on the Basis of NPE

According to the basic assumptions and current theoretical framework of Exercise Physiology, the physiological assessment of patients and athletes is traditionally focused on the evaluation of quantitative markers extracted from isolated variables and functions. Such markers provide little information about the coordinated activity and synergies of the physiological systems that are essential to generate behavior at the organism level and appear to not be sensitive and sufficiently responsive to training effects (Balagué et al., 2016; Garcia-Retortillo et al., 2019a), fatigue (Garcia-Retortillo et al., 2017), or nutritional interventions (Esquius et al., 2019), as well as to the prevention and diagnosis of common dysfunctions among athletes (e.g., states of overtraining, injuries, etc.; Meeusen et al., 2013). The majority of investigations in Exercise Physiology utilize static measures (maximal, averages, and threshold values), and the dynamic component of physiological processes is neglected. The significance of gradual increase or decline of physiological parameters during exercise and the nonlinear effects they produce on physiological networks have not been explored from an Exercise Physiology perspective.

According to the interaction-dominant dynamics of neurobiological systems, an NPE approach may prove to have larger potential to evaluate the fitness and training states on a coordinative basis and inform more accurately about the risks of dysfunctions. In this line, the NPE-based assessment has two main objectives: (a) evaluation of physiological networks structure and dynamics, and their evolution in time during and after acute and chronic exercise, and (b) evaluation of the responsiveness of the physiological network interactions to exercise perturbations. The first objective may use connectivity, modularity, causality, and synergy measures (Tognoli, and Kelso, 2014; Varona, and Rabinovich, 2016; Pol et al., 2018), while the second objective would require the detection and quantification of adaptive properties of the physiological network (e.g., stability, metastability, instability, critical behavior and fluctuations, critical slowing down, flickering, and other phenomena such as hysteresis and relaxation time after perturbation) that can be used as coordinative markers of interactions among systems during exercise-related states and functions (Hristovski et al., 2014).

The development of adequate technology of wearable devices, which are able to provide continuous and synchronous recordings (time series) of selected coordinative variables (order parameters) extracted from different physiological levels, is needed to study physiologic network dynamics. Computational intelligence methods, comprising algorithms inspired by nature (Fister et al., 2015) and robust methods able to infer couplings among diverse systems with different type of dynamics (oscillatory, multiscale, deterministic or stochastic, linear or nonlinear) that communicate with time-varying bursting activity (time delay stability; Bashan et al., 2012), could be successfully applied to design future functional evaluation tools based on NPE principles. Such algorithms can be implemented in modern mobile devices supplemented with EEG, EMG, and ECG sensors that are able to determine interorganic interactions during exercise testing. Until then, the availability of continuous recordings in laboratory settings of behavioral variables (e.g., extracted from kinematic or phenomenological data) as order parameters that contain integrated information of all physiological levels can be used to detect modularity in vertical and horizontal integration of the network.

### Current Limitations of Exercise Recommendations for Health and Disease

Principles of training derived from Exercise Physiology have remained largely impervious to the transdisciplinary and holistic insights emanating from complex systems approaches (Pol et al., 2018; Fullagar et al., 2019). In this section, the practical perspectives derived from the change of theoretical assumptions and research directions of NPE are illustrated through an example of exercise recommendations. Limitations of current guidelines of exercise prescription in health and disease are reviewed on the basis of NPE with the purpose of contributing to provide safer and more effective practical issues.

Physical activity is taking on an increasingly key role in the prevention and treatment of multiple chronic diseases, health conditions, and their associated risk factors. Despite its well-known health benefits, physical inactivity is considered a global pandemic and has been identified as one of the four leading contributors to premature mortality (American College of Sports Medicine, 2009). Governmental, academic, and research institutions: international organizations; sport associations; and the private sector recommend physical activity as part of a healthy lifestyle and for the prevention and treatment of a long list of chronic diseases. Overall, strong scientific evidence demonstrates that, compared to less active adults, individuals who are more active have lower rates of all-cause mortality and exhibit a higher level of cardiorespiratory and muscular fitness (Green et al., 2008; Wilson et al., 2016). New advances in precision medicine research show the beneficial effect of regular exercise at molecular, cellular, and whole-body levels (Friedenreich et al., 2016). However, there is limited research understanding exercising individuals as networked embedded systems and a clear absence of knowledge regarding the effects of exercise on the interactions among physiological systems.

Almost all studies testing the benefits of exercise for healthy persons and clinical patients closely adhered to the exercise guidelines of World Health Organization (2019) and American College of Sports Medicine (American College of Sports Medicine, 2009). Overall, the recommendations are similar for both healthy and clinical populations with few adaptations in function of the age and type of disease. The situation is not different in sports performance domain, where training recommendations for “optimal” (e.g., maximal) adaptation are proposed (Mujika et al., 2018).

Aerobic activity and strength training, prescribed as basic medication, are the core of programs addressed to healthy persons and clinical patients. Other type of activities related to flexibility such as Yoga or Pilates, which allow the improvement of range of motion and balance, are considered complementary. The recommendations are similar for Alzheimer’s disease, aneurysm, asthma, atrial fibrillation, bleeding disorder, blood lipid disorders, cancer, chronic kidney disease, chronic liver disease, chronic obstructive pulmonary disorder (COPD) depression or anxiety, heart failure, heart valve disease, HIV/AIDS, hypertension, fibromyalgia, inflammatory bowel disease (IBD), low back pain, mobility limitations, osteoarthritis, osteoporosis, overweight/obesity, pacemaker, Parkinson’s disease, peripheral arterial disease, prediabetes, pregnancy, rheumatoid arthritis, and Type 2 diabetes. Small load adjustments, in combination with some complementary practices, are recommended according to the specific disease.

Aerobic exercise, either alone or in combination with resistance training, at a moderate intensity (50–75% of a predetermined physiological parameter, typically age-predicted heart rate maximum or reserve), performed in two to five sessions per week with bouts equal or higher than 10 min is the general recipe. Ten to sixty minutes per session, with the ultimate objective of achieving at least 150 min/week is the minimum exercising time. Because of the assumed dose-response relationship, for more intense outcomes, the exercising time can be extended to 300 min/week or reduced to at least 75 min/week if the exercise has a more vigorous intensity. Strengthening activities performed at moderate or high intensity and involving all major muscle groups and practiced 2 days a week provide additional health benefits.

Despite the adoption of a relatively homogeneous prescription approach, aerobic and strength training have been, for the most part, associated with benefits across a diverse range of populations (e.g., Bishop et al., 1999; Voisin et al., 2015; Flannery et al., 2019; Klil-Drori et al., 2020; Maestroni et al., 2020). On this evidence, it is assumed that a standardized, largely homogeneous exercise prescription that adopts a conventional approach is safe, efficacious, and therefore sufficient.

Even though most studies present favorable results, systematic reviews and metaanalysis on exercise prescription point to the lack of high-quality studies showing the sustainability of standardized programs (Sørensen et al., 2006) and the need for personalizing the recommendations (Zimmer et al., 2018). The dominance of research based on comparisons of group data means evaluating quantitative changes of isolated variables in lab conditions is clearly limiting the application of a precision exercise medicine approach (Balagué et al., 2020).

Individuals respond and evolve in distinctive ways to standardized training programs (Mann et al., 2014), showing patterns of variability that are not captured by models based on statistical averages. Which people undergo positive effects? Average values mask inter-individual differences ‐ while some individuals respond with big positive effects, others have even detrimental effects. In addition, the attention in most evidence-based medicine and in particular in physical fitness research is almost exclusively restricted to inter-individual variations, neglecting intra-individual time-dependent variations (within each individual) which are better captured through time series recordings (Rose et al., 2013). As there is no equivalence between inter‐ and intra-individual variability, implementing precision medicine to exercise prescription requires focusing on this neglected time-dependent variation within single individuals. Only such recursive techniques allow personalizing treatments in place and time (Molenaar, 2004; Nesselroade and Molenaar, 2010).

The validity and reliability of tests based on inter-individual variability cannot be generalized to individual assessments of non-stationary processes like training and, thus, cannot provide the basis for individual counseling. The problems related to the biological and measurement variability of gold standard fitness markers like VO_2 max_, used in the evaluation of aerobic programs, have been widely discussed (Beltz et al., 2016). In fact, new variables of study, based on the covariation of time series of cardiorespiratory variables, have shown more sensitivity to training interventions than VO_2max_. (Balagué et al., 2016; Garcia-Retortillo et al., 2019a).

Another problem of current research on exercise prescription is derived from the bench to bedside approach. Numerous physical and social environmental factors affect fitness and health (World Health Organization, 2019) and the application of exercise programs tested under lab conditions may have a very different impact in real contexts. Motor actions emerge from the individual-environment interaction and, systematically, different patterns will emerge for different individuals (Araújo and Davids, 2016; Pol et al., 2020). Assuming that the behavior is stable, the available experimental models ignore the influence of context, focusing on averages to reveal “true” behavior. An 18-year longitudinal study has shown that comparable amounts of physical activity can lead to different effects on fitness or health status and have underlined the importance of contexts, content, and purposes of physical activity when health or fitness benefits are addressed (Schmidt et al., 2017). Accordingly, adaptation, not only from person to perso, but from moment to moment in space and time, is of utmost importance to produce effective results (Sturmberg, 2019).

Although systematic reviews and metaanalyses indicate that exercise therapy following a generic prescription is safe, tolerable, and efficacious (at improving symptom control outcomes), caution is recommended when interpreting these data: (1) the effects of exercise therapy are usually compared against a non-intervention control group with a sedentary lifestyle of recognized deleterious consequences, (2) metaanalyses and systematic reviews do not reduce but may enlarge the bias of studies that compound it (Weir et al., 2016).

Finally, research methodologies may also need to be improved to contribute to the development of personalized recommendations. The use of statistical inference techniques without enough criteria in available fitness research has produced a false belief that a significant result reflects the reality (Hristovski et al., 2017). This belief has led scientists and journal editors to privilege statistically significant results, thereby distorting the literature and leading to wrong interpretations (Wasserstein and Lazar, 2016; Amrhein et al., 2019). In research, when the posed question is wrong, multiple pathways cannot be detected initially because the alternatives are invisible to statistical techniques that rely on averages to characterize individual responses (Rose, 2016). Finally, systematic reviews do not solve the problem but may even make it worse because, rather than eliminating the bias, they compound it (Weir et al., 2016).

The significant benefit of a generically dosed exercise on heterogeneous populations reflects the remarkable pleiotropic physiological impact of exercise. In this sense, it is warranted to investigate further the therapeutic properties of exercise medicine to reveal its whole potential role in healthcare. Although it is still not known whether alternative prescriptions adopting a more personalized approach will confer superior efficacy to exercise treatments, several efforts have pointed toward this direction (Jones et al., 2017). In fact, exercise scientists are continuously exploring the tenets of performance to refine and personalize exercise training with the aim of minimizing injury and maximizing benefits.

### Some Hypotheses for Future Applicative Research

Due to safety reasons, the current standardized training programs addressed either to healthy individuals, clinical patients, and athletes require the controversial quantification of the relative exercise intensity (Jamnick et al., 2020). And, accordingly, the use of ergometers and strength machines. These technical devices, mostly found in fitness facilitie where healthcare providers and trainers use to refer their users/patients/athletes, reduce the physical activity to cyclic (walking, running, stepping or cycling) and repeated local body movements. In such context, diversity is mostly minimized to volume and intensity changes. Although varying the volume and intensity of exercise increases the diversity potential of individuals, varied activities in natural environments, highly recommendable (Brymer et al., 2020) may help to enhance further this potential. As pointed by Hristovski and Balagué (2012), such open-air activities (mountain climbing, swimming in the sea, etc.) embed performers in a multi-time-scale fluctuation regime of resistance that provides high adaptive effects on body functions. Accordingly, the authors propose to implement in exercise machines (e.g., cycle ergometers, rowing machines, vibrating platforms, steppers, etc.) a multi-time-scale stimulator system to manipulate the variability dynamics and simulate the fractal dynamics found in nature.

In fact, the growing number of fitness specialties (46 in the 2020 ACSM’s Worldwide Health and Fitness Trends Survey) is a proof that many types of activities, not only those based on cyclic or repetitive movements, may contribute to fitness and health development. In particular, those activities chosen by users/patients/athletes and intrinsically motivating, if adequately adapted by an exercise professional (introducing progressively new challenges), may favor the adherence to practice (Balagué et al., 2020).

Intelligence in CAS has been recently described as a tendency to evade and escape states of reduced fitness, that is, states of reduced functional diversity potential (Hristovski and Balagué, 2020). This refers to richness of functional synergies and also fast recovery time after a perturbation (see hysteresis behavior of section CAS Interact Dynamically and Non-linearly, i.e., Co-Adaptively, With the Environment). The intelligent behavior may be expressed at diverse levels. For instance, bouts of exercise produce acute fatigue that temporarily decreases the diversity potential of the organism. However, the cell or organism reacts by a temporary increase of the diversity potential anticipating the possible future incoming perturbations. These types of biological behaviors have been modeled as strong anticipation phenomena (Dubois, 2003; Stepp and Turvey, 2010). These exercise effects can particularly compensate the tendency of aging and disease to reduce the diversity potential (Yogev et al., 2007; Rutenberg et al., 2018).

The growth of intelligence requires regular coupling to challenging and stimulating environments to evade the temporary stalemate, which may, on longer time scale, turn into decreasing functional diversity potential (Hristovski and Balagué, 2020). On the other hand, the diversity potential can be reduced due to unexpected perturbations (e.g., the pandemia effects) and the property of biological intelligence is to escape quickly from it through the creation of new synergies which may include new dimensions, not only those related to exercise modalities. In fact, exercise is not the only intervention that may increase the functional diversity potential and/or evade its reduction. Healthy diet, stress reduction, inspiring intellectual work, music, art, meditation, etc. may all contribute to it.

As health and fitness have also subjective dimensions (Sturmberg, 2019), they can be satisfied in multiple idiosyncratic personal ways. This means that the individual satisfying diversity potential is also associated to a subjective experience of wellbeing, and this experience can be recovered in many different ways due to available multidimensional compensations.

Some long-lasting personal or environmental constraints may produce a cascade of long-lasting effects on physiological levels (Balagué et al., 2019) and the response to physical activities may change according to it. Due to the multidimensionality, context-dependency and subjectivity of health and fitness, a personalized exercise recommendation may prove to be more adequate than current standardized exercise programs addressed either to healthy individuals or clinical patients.

It may become recommendable to reorient the main aim of prescribed exercise medicine toward gaining diversity through the development of multidimensional and multiscale synergies. Exercise dosage and formulation, as occuring in personalized medicine, may be adapted accordingly. The formula of an active life with varied stimulus, preferably at open air (Ryan et al., 2010), would then be shown to be more adequate than reducing the physical activity to 75–150 min/week repeating exercises.

As synergy formation is better captured through the interactions among the involved components and processes, variables related to connectivity (number and strength of couplings) can be suitable to test the exercise program effects. Because components of the network cooperate to accomplish the common fitness goal, if the number and strength of couplings is reduced, other components of the network may become overwhelmed and the system may respond less effectively to perturbations, bringing about dysfunction and increased susceptibility to injuries (Pol et al., 2018).

Exercise, in turn, having a profound impact on human metabolism (Koay et al., 2020), may produce very relevant perturbations on the network dynamics at multiple levels (see Figure 1), and thus, provide an accurate information about its resilience and antifragility of the organism, key properties to inform about its state. In summary, the NPE approach may transform not only the criteria for exercise prescription but also the comprehension of other fields of knowledge, typically studied under the framework of Exercise Physiology like functional evaluation, injury prevention or limits of performance. It also may provide a new understanding of many exercise-related phenomena as fatigue, overtraining, injuries, etc. currently influenced by a reductionist scientific approach.

## Conclusion

Current Exercise Physiology, deeply influenced by reductionism, is limiting the understanding of exercise-related phenomena and hampering practical applications to sports performance and exercise as medicine. Integrative Exercise Physiology approaches, methodologically based on statistical inference techniques and focused on timeless vertical, bottom-up mechanistic causation (from the sub-cellular and cellular levels to organ and systemic functions), are not sufficient to improve substantially the current state of physiological research.

Inspired by the new field of Network Physiology and Complex Systems Science, NPE emerges to transform the theoretical assumptions, the research program and the current practical issues of current Exercise Physiology. It focuses the research efforts on improving the knowledge of the nested dynamics of the vertical network interactions and, particularly, the horizontal integration of key organ systems. Through the application of novel methods and approaches derived from recent advances in Network Theory, Nonlinear Dynamics, Computational and Statistical Physics, and Biomedical Informatics, it seeks to provide insights into Basic Physiology itself as well as for Exercise Physiology.

The critical view on the current one-size-fits-all approach of exercise prescription in health and disease, in contrast with a new proposal based on complex systems and NEP principles, illustrates the potential practical impact of the approach, which aims to provide: (a) a theoretical framework to address problems and challenges in Network Physiology, and particularly, in NPE, (b) data-driven discoveries of the basic physiological laws and control mechanisms that underlay network interactions for various states under both healthy and pathological conditions with focus on Exercise Physiology and Sports Medicine, and (c) a forum for developing new methodologies, a vision and a programmatic approach on applications of NPE. In this fashion, more qualitative research directions in Exercise Physiology may be developed and an original and fertile research program can emerge in the near future.

## Data Availability Statement

The original contributions presented in the study are included in the article/supplementary material, further inquiries can be directed to the corresponding author.

## Author Contributions

All authors listed have made a substantial, direct and intellectual contribution to the work, and approved it for publication.

### Conflict of Interest

The authors declare that the research was conducted in the absence of any commercial or financial relationships that could be construed as a potential conflict of interest.
